# Educational Needs Regarding Mental Health of Professionals Working with Young Adults with Cancer: A European Survey

**DOI:** 10.3390/curroncol33050263

**Published:** 2026-04-30

**Authors:** Sid Morsink, Leonieke W. Kranenburg, Johanna Berg, Evangeli Bista, Saintuya Dashondog, Siret Kivistik, Mari Lahti, Carmen Monge-Montero, Joaquim Oliveira Lopes, Evanthia Sakellari, Duygu Sezgin, Margarida Rodrigues Tomás, Merle Varik, Wendy H. Oldenmenger

**Affiliations:** 1 Department of Psychiatry, Erasmus MC, University Medical Center Rotterdam, 3000 CA Rotterdam, The Netherlands; 2 Turku University of Applied Sciences, 20520 Turku, Finland; 3 Kentro Kathodigisis Karkinopathon Kapa 3, 115 25 Athens, Greece; 4 Health Promotion Research Centre, School of Health Sciences, University of Galway, H91 TK33 Galway, Ireland; 5 Tartu University Hospital, 50406 Tartu, Estonia; 6 Tartu Applied Health Sciences University, 50411 Tartu, Estonia; 7 Youth Cancer Europe, 400372 Cluj-Napoca, Romania; 8 Nursing Research, Innovation and Development Centre of Lisbon (CIDNUR), Nursing School of Universidade de Lisboa, 1600-190 Lisboa, Portugalmatomas@enfermagem.ulisboa.pt (M.R.T.); 9 Department of Public and Community Health, Laboratory of Hygiene and Epidemiology, University of West Attica, 11521 Athens, Greece; 10 School of Nursing and Midwifery, University of Galway, H91 TK33 Galway, Ireland; 11 Department of Medical Oncology, Erasmus MC Cancer Institute, University Medical Center Rotterdam, 3015 GD Rotterdam, The Netherlands; w.h.oldenmenger@erasmusmc.nl

**Keywords:** young adults with cancer, mental health, screening, psychosocial support, educational needs

## Abstract

**Background:** Young adults (YAs) with a cancer diagnosis are at an increased risk of experiencing mental health problems after cancer treatments. Evidence underlines the benefits of providing psychosocial cancer care as a part of standard care in reducing distress and psychological morbidity. The objective of this European study was to identify the educational needs of Health Care Professionals (HCPs) regarding mental health screening and psychosocial support for YAs with cancer. **Methods:** An international, cross-sectional online survey was conducted to examine current practices and priorities for future training. The target population was European HCPs involved in the care of young adults with cancer. The survey was disseminated through national and European platforms. **Results:** In total, 271 HCPs completed at least 70% of the survey. Respondents came from 21 different European countries. Around half of the participants thought they could screen for distress, anxiety, and depression and tended to do so; however, only 10% of them used validated screening tools for this purpose. Most respondents (70%) said that they needed training on the use of screening tools for mental health problems. Training in recognizing (the early signs of) cancer-related mental health problems was also prioritized. **Conclusions:** The results show that HCPs in Europe could improve their daily practice regarding the recognition and screening of mental health issues in young adults with cancer. HCPs particularly reported the need for future training in using screening tools.

## Simple Summary

Young adults with cancer have a higher risk of mental health problems after treatment. Psychosocial care can help reduce distress and improve well-being. This European study examined the training needs of healthcare professionals (HCPs) regarding mental health screening and support for young adults with cancer. An online survey was completed by 271 HCPs from 21 European countries. About half of the participants believed they were able to screen for distress, anxiety, and depression, but only 10% used validated screening tools. Most respondents (70%) reported needing training in how to use these tools. Many also wanted more training in recognizing the early signs of cancer-related mental health problems. The results show that mental health screening practices could be improved and that HCPs prioritize further education, especially in using proper screening tools.

## 1. Introduction

Worldwide, an increase in cancer diagnosis has been identified, especially in people under 50 years and in young adults (YAs, age group 18–39 years) [1]. Getting cancer during young adulthood can severely disrupt emotional, cognitive, and social development [2]. As a result, young adults with cancer and YA survivors can encounter significant mental health challenges throughout their cancer journey, which differ from those experienced by children and older adults [3]. Their challenges typically involve identity, career, and family planning [4]. Also, the influence of cancer on their cognitive and physical development, body image, and autonomy negatively affects their health-related quality of life [5]. 

Young adult cancer survivors face an increased risk of developing mental health problems after completing treatment. For example, survivors of childhood, adolescent, and young adult cancers are 57% more likely to experience depression and 29% more likely to develop anxiety in the years following treatment, compared to their siblings or healthy individuals [6]. Integrating psychosocial care into standard oncology practice at different stages of the cancer journey helps reduce distress and psychological morbidity, improves quality of life during and after treatment, and might even contribute to increased survival [7,8,9].

Yet, mental health concerns in the YA population are often poorly recognized by health care professionals (HCPs) [10], who may view expressions in this regard as natural reactions to a cancer diagnosis. Furthermore, HCPs have been reported to lack the necessary skills and resources to detect early signs of mental health problems and to respond appropriately [11]. This may be further complicated by the fact that care for YAs with cancer often falls between child and adult cancer care, leaving the age-specific needs of this group unmet [12,13]. For instance, the study by Rutkowski et al. (2026) showed that only about half of pediatric and adult cancer care sites in their study reported offering any YA-specific cancer services or programming [13]. As a result, providing adequate psychosocial support by HCPs that meets the mental health-related needs of YAs with cancer can be challenging [4,14]. 

Although the educational requirements for training on mental health-related topics in YAs with cancer are not well defined so far, the educational needs of HCPs working with YAs with cancer have been widely recognized in the literature. Several studies highlight the importance of ongoing education for HCPs in this field [14,15,16]. Bradford et al. [16] reported a shift in the predominant educational needs over the past decade. Initially, educational demands focused primarily on palliative care and biomedical topics. Over time, these were supplemented by increasing requests for psychosocial and practical training, including education on fertility, sexuality, and the management of late effects. These findings align with the support needs identified by YAs themselves, such as fertility preservation, sexual health, return to school or work, and management of cancer-related fatigue [17].

Over the past decades, the specific needs of YAs with cancer have become both more acknowledged and more pronounced. A recent large international consensus initiative, including adolescents and young adults with lived experience of cancer, caregivers, health-care professionals, researchers, and policy makers has arrived at a core outcome set for YAs with cancer, to be used in clinical care and for research purposes [18]. This outcome set consists of 20 outcome measures, acknowledging “Access to mental health support”, “Psychological distress” and “Fear of cancer recurrence (off-treatment only)” among the most important outcomes. This clearly illustrates the importance of appropriate mental health care in this group. Notably, mental health is important in itself, but is also clearly related to other domains identified as core outcomes. For example, the outcomes of “Pain” and “Fatigue” are mutually affecting “Psychological distress”. In addition, financial strains, in the outcome set defined as “Financial toxicity” may lead to significant and diverse shortages, all leading to increased psychological distress. 

Current interventions addressing the needs of YAs with cancer primarily focus on physical well-being, psychological well-being, social well-being, and survivorship care [17]. Within the domain of social well-being, a relatively new approach is Social Prescribing, a community-based intervention designed to connect individuals with activities and services that address health and social care needs. Early evidence suggests that Social Prescribing may improve health-related outcomes in cancer survivors, including increased confidence, enhanced mental health, and reduced fatigue [19]. Another emerging intervention aimed at improving both physical and mental health involves increased engagement with green and blue spaces [20]. Studies indicate that exposure to natural environments can support coping with cancer and its aftermath and may contribute to both physical and psychological recovery among cancer survivors [18,19,20]. 

To advance and innovate care for YAs with cancer, this study primarily seeks to identify the educational needs of European healthcare professionals regarding mental health screening and the provision of psychosocial support for this population. Secondarily, it investigates how emerging psychosocial support modalities—particularly the use of green and blue spaces and Social Prescribing—inform and shape these educational needs. 

## 2. Materials and Methods

This study was conducted as part of the European MELODIC consortium, led by Turku University of Applied Sciences in Finland. The overall aim of the MELODIC project is to promote the mental health and well-being of young adults with cancer in the first years after cancer diagnosis. 

Study design: An international, prospective, cross-sectional online survey was employed to identify the educational needs of HCPs related to mental health screening, detection, and the support of YAs with cancer. The international research consortium, consisting of more than twenty experts in the field of oncology, mental health, and public health, developed the survey based on the literature [10,11,12,13,14,15,16,17,18,19,20,21,22,23] and the clinical expertise of the international consortium members (see Appendix A). Reporting is in line with the Consensus-Based Checklist for Reporting of Survey Studies (CROSSs) [24]. 

Participants: The target population were all European HCPs involved in the care of YAs with cancer, including physicians, nurses, health visitors/community health scientists, psychologists, etc. Potential participants could consent to participate after reading the participant’s information sheet. The online survey was anonymous, and participants were only asked to answer questions about general, non-identifiable clinical practices in their region.

Recruitment procedures: Data were collected between May and August 2025. The survey was designed using Castor, a web-based data collection platform. First, the survey was piloted to check its functionality. Participants were invited to participate in the survey through emails, newsletters, and social media posts from the MELODIC consortium partners and European, national, and local platforms for HCPs working in cancer care. This introduced potential participants to the study and directed them to the consent form. A general anonymous link to the online survey was provided. Potential participants were able to read information about this study and to give consent before starting the survey.

Data collection: Participants were asked to complete basic demographics, including their age, gender, residential country, profession, work setting, and years of experience in work with YAs with cancer. The survey examined their current practices and their needs and priorities for future training. Respondents were asked whether they felt capable of performing thirteen distinct professional activities related to mental health screening and support with special attention to the use of green and blue spaces and Social Prescribing. The respondents valued their capability on those activities on a five-point scale (I think I can do this: not at all—not sufficient—more or less—sufficient—good). Consequently, they were asked whether they actually performed these activities in practice (I do this: never—rarely—occasionally—frequently—always). See also Appendix A. 

To explore HCPs’ educational needs, we asked their appreciation on nine suggested topics for future training, reporting the necessity on a five-point scale (definitely not—probably not—maybe—probably yes—definitely yes). The topics were: receiving general information about mental health in young adults with cancer; training in using screening tools for mental health problems; training in addressing mental health during consultations; training in addressing social relationships; training in recognizing cancer-specific mental health problems; training in recognizing early signs of mental health problems; training in using green and blue spaces (natural places such as parks, forests, beaches) to improve mental well-being; training in using physical activity to improve mental health; and training in using Social Prescribing. Additionally, the respondents were asked to select the three educational training suggestions that would be most helpful to them. 

Data were centrally collected and stored by one of the consortium centers (Erasmus MC, the Netherlands). Ethical approval for data collection and storage was obtained by the Ethical Review Board of the Erasmus University Medical Centre (6 February 2025). 

Data analysis: Data were analyzed in IBM SPSS Statistics for Windows (Version 29.0.2.0). The analysis proceeded using descriptive statistics for the demographics of the participants. To analyze the current practices for the surveyed thirteen distinct professional activities, HCPs’ self-reported capabilities were defined as sufficient when they ranked them as ‘sufficient’ or ‘good’. When the practice of an activity was ranked as ‘frequently’ or ‘always’, it was defined as a frequent part of HCPs’ daily practice. To analyze the educational needs for future training, we defined a professional activity as an HCP’s need, when a topic was ranked with ‘probably yes’ or ‘definitely yes’ by the participants. 

## 3. Results

### 3.1. Demographics 

In total, 271 HCPs completed the survey for at least 70%, and 183 HCPs completed all items of the survey. Demographic characteristics are described in Table 1. The typical respondent was an HCP > 45 years old (46%), a female (85%), a nurse (40%) or a medical doctor (26%), working in a public cancer center (31%) or a public university hospital (31%), and had over 10 years of work experience (58%). Respondents came from 21 different European countries. 

### 3.2. Current Practices 

**Figure 1:**
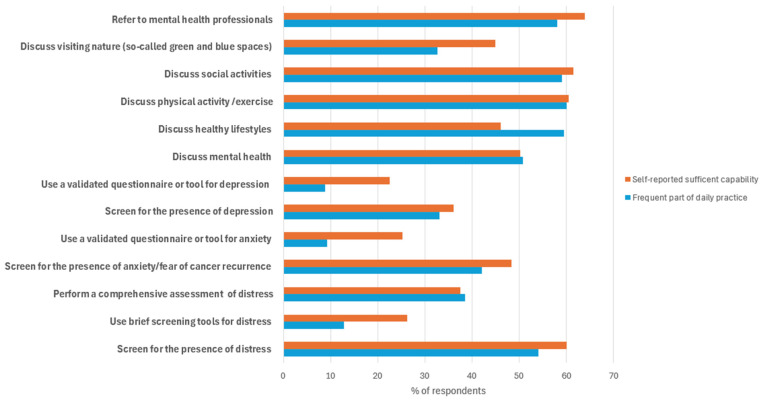
HCPs’ self-reported capabilities and current daily practices of professional activities regarding screening and supporting of mental health issues in young adults with cancer.

Two hundred and five respondents completed the section of the questionnaire regarding their current practices for screening mental health issues and providing psychosocial support. The results regarding current practices are illustrated in Figure 1. 

Overall, 60% of the respondents believed they can screen for the presence of distress in YAs, and 54% did this regularly in their daily practice. While a quarter of respondents thought to be able to use brief screening tools, only 13% actually did so. Thirty-eight percent of the respondents perform a comprehensive assessment in case someone screens positive for distress. 

Around half of the respondents thought they could screen for anxiety in YAs, and 42% of them indicated they did. However, only 9% used validated questionnaires or tools for this purpose. Regarding depression, approximately one-third of the respondents believed they could screen for it in YAs, and they did so in their daily practice. Twenty-three percent of the respondents thought they could use validated questionnaires or tools for depression screening, but only 9% actually did so. 

Half of the respondents believed that they discuss mental health sufficiently and discussions were performed frequently by half of the respondents. Sixty-four percent of the respondents thought they could refer YAs to mental health professionals, and 58% indicated they did so. 

Discussing healthy lifestyles and discussing physical activity were a frequent practice among 60% of the respondents. The use of green and blue spaces was discussed by a much smaller number of respondents (33%). About 60% of the respondents thought they were sufficiently capable of discussing social activities with YAs and the same number tended to do so in their practice. 

### 3.3. Educational Needs and Priorities 

**Figure 2:**
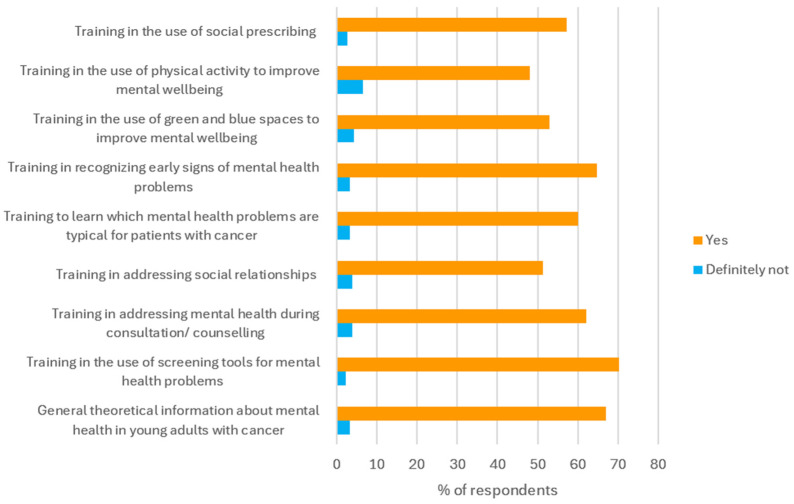
Educational needs for future training by HCPs working with young adults with cancer, regarding 9 professional activities on mental health screening and support. Yes = ‘probably yes’ or ‘definitely yes’.

A total of 185 respondents answered the question about their educational needs. Regarding all the suggested topics for improving their skills in mental health screening and support, between 48% and 70% of the respondents reported that they need additional training. Very few respondents (2.7–6.5%) thought they definitely did not need this kind of training. Most respondents (70%) said they needed training on the use of screening tools for mental health problems (Figure 2).

**Figure 3:**
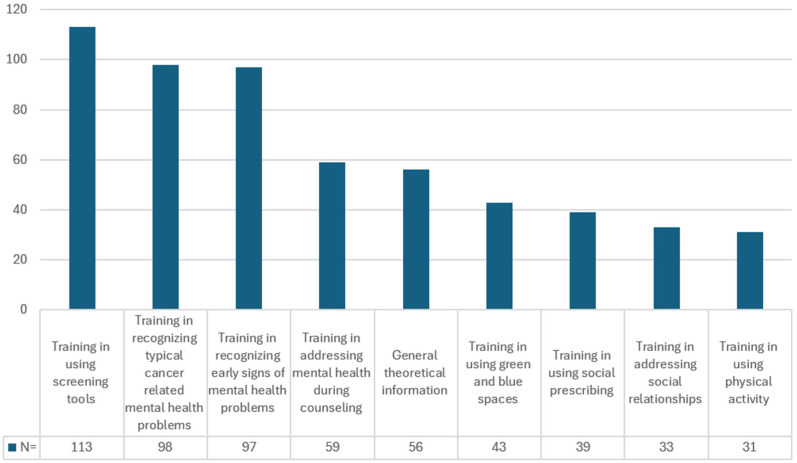
Priorities in topics for future training by HCPs (top 3 ranking).

The three topics that were ranked as the highest priority are related to recognizing and screening for mental health issues, with the use of screening tools being mentioned the most among HCPs (113 participants). Training in addressing social relationships and using physical activity were the two least frequently prioritized topics (Figure 3). 

## 4. Discussion

The aim of this study was to identify the educational needs of HCPs regarding mental health screening and psychosocial support in the care of young adults with cancer. This survey shows that, in the daily practice of many European health care providers in young adult cancer care, there is a challenge in the screening for, and recognition of mental health complaints. In particular, the structured use of a screening tool is something that only a limited number of respondents reported doing. Low use of screening tools has been previously described in support consultants in oncology [25]. They reported that using a screening tool does not always feel appropriate in practice, which may also play a role in our study sample of HCPs. Furthermore, 85% of the HCPs in this study were female. The female gender of an HCP positively affects the patient-centered dialog on the psychosocial situation of the patient [26]. This may result in a lower felt urge to use a screening tool to promote this dialog.

At the same time, the use of screening tools to recognize mental health problems in young adults with cancer was also rated as a top priority for future training by the HCPs participating in this study. This converges with the study of Kim et al. (2021) [27] with regard to training contents, as the HCPs in their study were also most interested in learning about symptoms and pathways, possibly indicating the HCPs wish to act in time and prevent an escalation of mental health problems, as is recommended by the NCCN Adolescent and Young Adult Oncology Guidelines [28].

The existing literature on the educational needs of HCPs specifically focusing on mental health in YAs with cancer is limited and mostly restricted to local contexts or patient groups [14,29]. For example, HCPs working with YAs who have an uncertain or poor cancer prognosis have been found to require specific education, as caring for this population has been associated with significant emotional and practical challenges. HCPs may experience heightened emotional involvement and difficulty communicating about prognosis and end-of-life issues. Educational interventions aimed at supporting professionals’ own well-being and improving communication skills are therefore essential [14]. 

On a broader level, our results suggest that it would be beneficial to develop certain standards of education for professionals who work with adolescents and YAs with cancer. A recent review (2026) in fact underlined the importance of education for HCPs working with YAs with cancer. This review aimed to capture relevant clinical practice guidelines on core elements that define quality care for YAs with cancer [30]. Eight clinical practice guidelines for the care of YAs with cancer, mostly from prominent oncology guidelines bodies, were included [31,32,33,34,35,36,37,38]. Taking all the guidelines together, 13 joint themes could be identified. One of these was “Raising awareness” which included the importance and recommendation of professional education and training education for healthcare providers. For instance, the ESMO-SIOPE position paper [35] states that “Increasing awareness of YA-related cancer and educating healthcare providers, as well as the patient and their families has been recognized […] as being of utmost importance for the optimal delivery of holistic cancer care for YAs.”

Also, based on this guideline assessment, recommendations were made with regard to the theme of “Psychosocial and Developmental Support”. In line with the results of the current study, the development and use of age-appropriate screening instruments was explicitly named. We agree with the TCT recommendations [34], that screeners can also serve to elicit meaningful conversations with YAs with cancer on their psychosocial needs. Many mental health screening instruments are not YA-specific, let alone YAs with cancer-specific. An exception is the Adolescent and Young Adult Psycho-Oncology Screening Tool (AYA-POST) [39]. This instrument is cross-validated with a common measure for anxiety and depression, the Hospital Anxiety and Depression Scale [40] along with the cancer-specific Distress Thermometer [41] in a YA-population, yielding an easily applicable tool to assess psychosocial well-being in YAs with cancer [42]. Newer initiatives include the EORTC Health-Related Quality of Life Tool for Adolescents and Young Adults With Cancer [43]. Pilot testing of this questionnaire resulted in 30 items covering five subscales (activity limitations and life disruptions, worry about cancer and the future, self-esteem, relationships, and positive changes) and nine single questions. It was found that items on distinguishing between important and nonimportant things in life, and on motivation to live life to the fullest were considered most relevant. 

We found that among our participants, there appears to be less need for training in specific interventions aimed at promoting mental health, in particular the use of Social Prescribing or physical activity. This may be because these interventions are not considered as “interventions” per se, but as “routine care” which is already being offered. Indeed, we found that many HCPs indicated that they felt confident in this respect and often discussed the benefits and options of social and physical activities with YAs with cancer. However, regarding the benefits of green and blue spaces, we found low training needs despite the finding that only 30% tended to discuss this topic regularly. Another explanation for educational needs regarding recommending the use of green and blue spaces may be that this is a relatively new development, and its potential benefits for YA with cancer are still underrecognized [20]. 

Strengths and limitations: To our knowledge, this is the first study to map the educational needs of HCPs working with YAs with cancer at the European level. Although the sample size is relatively small, and subgroup analyses were therefore not feasible, this study illustrates that attention to the mental health of YAs with cancer extends beyond national health care contexts. As national health systems differ throughout Europe, generalizing our results to all European countries may be limited. A more specific analysis with a larger number of respondents per country would lead to a more profound understanding of how local organization of care influences HCPs’ educational needs.

The results of this survey study provide insight into how perceived barriers described in the existing literature [14,29] can be translated into educational needs for future training. The subject of addressing mental health needs, in general or specifically in patients with cancer, may have been covered differently in the various curricula in different health- and medicine-focused studies across Europe. This makes it difficult to make comparisons. Therefore, the respondents were asked to indicate what they consider important and how competent they feel in these areas—which may reflect their daily practice more appropriately than what was ever taught during their training. Still, larger numbers of respondents with equal representation of several professions involved in YA cancer care, and information about their qualifications and education level, would improve recommendations even more for future training for specific professions. 

In general, the use of a validated questionnaire is preferred to gain reliable results. However, no validated questionnaires for the specific topic of our study were available. Nevertheless, the questionnaire developed for this study enabled us to gain valuable insights into daily practice and the educational needs of HCPs and was feasible to use in different European countries. Further development of an extensively validated questionnaire on these topics could be of interest for the future. We addressed Social Prescribing and the use of green and blue spaces as modalities to investigate the position of emerging psychosocial interventions in HCPs daily practice and educational needs. Although this may not supply an entirely comprehensive insight into the use of emerging mental health initiatives in YAs with cancer, it may direct future development of HCP training in using evidence-based interventions, supplementary to adequate screening skills.

Clinical implications: The results of this study underscore the importance of training in screening skills for mental health among HCPs working in YA cancer care. They play a crucial role in identifying mental health challenges at an early stage. Given that evidence-based treatments exist for this population, it is essential to ensure screening, early identification, and timely access to effective psychosocial interventions [44]. Educating HCPs in the specific mental health needs of YAs with cancer, and how to detect these, would equip HCPs to detect problems sooner, respond appropriately, collaborate effectively with colleagues, and provide integrated support to this population affected by cancer. Our findings are in line with those of Kirchhoff et al., who advocate for targeted educational initiatives to enhance providers’ awareness of the broad impact of cancer on YA patients [15]. 

## 5. Conclusions

The results show that there is room for improvement in the daily practice of HCPs in Europe regarding the recognition and screening of mental health issues in young adults with cancer. This study is a first step in improving mental health support for YAs with cancer through developing adequate training for HCPs on a European level. HCPs prioritize training in using screening tools to improve their skills in early recognition of mental health issues in YAs with cancer. The use of innovative psychosocial interventions such as using green and blue spaces and Social Prescribing are, at this moment, less prioritized but can be a future direction for further research. 

## Figures and Tables

**Table 1:** Socio-demographic characteristics of HCPs participating in the survey.

		N = 271 N (%)
Age	<25 years	18 (6.6)
	25–34 years	47 (17.3)
	35–44 years	81 (29.9)
	45–54 years	84 (31.0)
	55–64 year	32 (11.8)
	>65 years	8 (3.0)
Gender	Male	37 (13.7)
	Female	230 (84.9)
	Non-binary	1 (0.4)
	Do not want to disclose	2 (0.7)
Country	Greece	56 (20.7)
	France	46 (17.0)
	Portugal	38 (14.0)
	Ireland	28 (10.3)
	Estonia	24 (8.9)
	Netherlands	23 (8.5)
	Other	56 (20.7)
Work setting	Public cancer center	85 (31.4)
	Public University hospital	84 (31.0)
	General public hospital	54 (19.9)
	Private cancer center	21 (7.7)
	Other	27 (10.0)
Profession	Nurse	109 (40.2)
	Medical Doctor	69 (25.5)
	Psychologist	23 (8.5)
	Health Visitor	21 (7.7)
	Social worker	9 (3.3)
	Community health scientist	4 (1.5)
	Spiritual/religious counselor	2 (0.7)
	Other	34 (12.5)
Work experience	<1 year	15 (5.5)
	1–5 years	56 (20.7)
	5–10 years	42 (15.5)
	10–20 years	87 (32.1)
	>20 years	70 (25.8)

## Data Availability

The original contributions presented in this study are included in the article. Further inquiries can be directed to the corresponding author.

## Abbreviations

The following abbreviations are used in this manuscript:

YA	Young adult
HCP	Health care professional
